# Recent Advances in Postharvest Irradiation Preservation Technology of Edible Fungi: A Review

**DOI:** 10.3390/foods12010103

**Published:** 2022-12-25

**Authors:** Yuanyuan Zhong, Shuting Dong, Yuan Cui, Xiaobo Dong, Huaide Xu, Mei Li

**Affiliations:** College of Food Science and Engineering, Northwest A&F University, Yangling, Xianyang 712100, China

**Keywords:** edible fungi, irradiation technology, preservation, quality, shelf life

## Abstract

Edible fungi have high edible, medicinal and economic value. Rapid development of the edible fungi industry can meet people’s consumption demands. However, due to lack of suitable preservation technology after harvest, edible fungi are susceptible to mechanical damage, microbial infection, and discoloration, which could affect the quality and shelf life of fresh edible fungi. Many techniques have been developed to extend the postharvest storage time of fresh edible fungi and irradiation technology has been proven to be one of the potential technologies. This review summarizes the internal and external factors affecting the postharvest quality deterioration of edible fungi, introduces the types of irradiation preservation technology and describes comprehensive advances in the effects of irradiation on shelf life, microbiology, organoleptic qualities, nutritional qualities (proteins, fats, sugars and vitamins) and enzymatic activities of edible fungi from different regions and of different species worldwide. This review uncovers that the postharvest quality decay of edible fungi is a complex process. The irradiation preservation of edible fungi is affected not only by the edible fungus itself and the storage environment but also by the radiation type, radiation dose and radiation source conditions. Future studies need to consider the combined application of irradiation and other novel technologies to further improve the preservation effect of edible fungi, in particular in the area of irradiation’s influence on the flavor of edible fungus.

## 1. Introduction

Edible fungi are large fungi that can form large fleshy (or colloidal) fruiting bodies or sclerotia-like tissues and can be used for food or medicine. More than 6000 species of edible fungi exist in nature, of which more than 2000 species can be used for consumption [1]. Nowadays, China is the world’s most important producer of edible fungi and 30 species of edible fungi have been industrialized, such as *Lentinus edodes*, *Auricularia auricula*, *Pleurotus ostreatus*, *Flammulina velutipes*, *Pleurotus eryngii*, *Agaricus bisporus* and *Agrocybe aegerita* [2,3]. The production of edible fungi in China reached 40.61 million tons in 2020 according to data from the China Edible Fungi Association, with the total production exceeding 80% of the world total and a value of more than USD 51.4 billion [4]. The reason for the increasing consumption of edible fungi is that they combine high medicinal value and nutritional characteristics as food. Relevant studies confirmed that edible fungi are not only rich in protein, essential amino acids, dietary fiber, vitamins and other nutrients [5], but also contain a variety of bioactive substances with certain efficacy, such as high molecular polysaccharides, polyphenols, triterpenes, etc. [6,7]. However, the commercial circulation of fresh edible fungi after harvest will face a major challenge: they are one of the most perishable products and often lose quality quickly after being harvested. Therefore, appropriate and efficient postharvest preservation methods are of great significance for improving the economic value of edible fungi.

**Figure 1:**
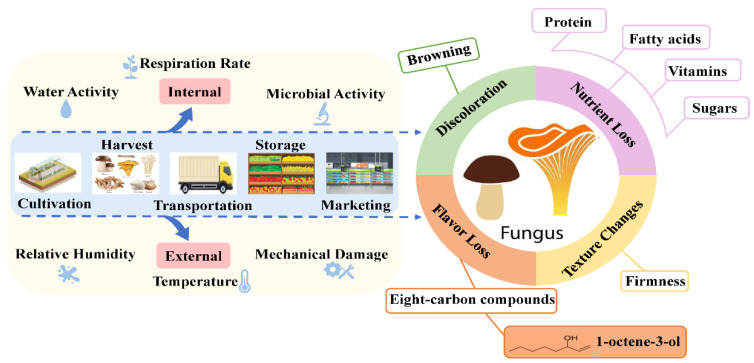
The relationship between postharvest quality degradation and influential factors.

The quality attributes of edible fungi after harvest are affected by variable factors, including internal factors related to edible fungi themselves (i.e., microbial activity, respiration rate and water activity) and external factors related to storage conditions (relative humidity, storage temperature and mechanical damage) (Figure 1). The tissue of edible fungi has a high moisture content (85–95%) [8], and the outer epidermis lacks protective tissue and loses a large amount of moisture under transpiration after picking [9]. Secondly, temperature fluctuation can enable edible fungi to activate a variety of oxidases, enhance physiological activity and increase postripening during storage [10,11]. In addition, edible fungi are vulnerable to damage, especially the invasion of pathogenic microorganisms during picking, transportation and storage [12]. In this regard, postharvest changes in edible fungi, such as cap opening, stipe elongation, cap diameter increase, etc. [13], further lead to discoloration [14], texture changes [15] and moisture loss [16] (pp. 203–211), thus reducing the edible and economic value of edible fungi and affecting consumers’ purchasing behavior.

There are extensive studies on developing and implementing preservation technology for edible fungi. Refrigeration [17], modified atmosphere packaging [18], coating [9], washing [19] and ozone treatment [20] are frequently applied technologies. However, they also produce adverse effects including contamination with pathogenic microorganisms, off-flavors, discoloration and safety considerations, and are unsuitable for application on an industrial scale [21]. In contrast, heating and ionizing radiation treatment can completely inhibit or inactivate microbial growth, resulting in producing shelf-stable food [22]. Although Fernandes, Antonio, Oliveira, Martins and Ferreira [21] reviewed the effects of gamma and electron beam irradiation on the physicochemical and nutritional properties of mushrooms before 2012, there has not been a recent detailed review on research advances in the area. Therefore, this review focuses on the recent developments of irradiation technology in edible fungus postharvest quality preservation, compares the preservation effects of different irradiation technologies and discusses the impact of irradiation treatment on edible fungi. This review aims to provide a theoretical reference for the study of the storage methods for prolonging the postharvest shelf life of edible fungi. Finally, some future research trends are proposed.

## 2. Irradiation Techniques and Species

Food irradiation is a recognized and established food processing technology worldwide, and another breakthrough after pasteurization technology. Irradiation sterilization processes food at or near ambient temperature, and is known as cold pasteurization and belongs to the non-thermal treatment processes [23]. Irradiation treatment is particularly important for producing shelf-stable agricultural products at ambient temperatures, such as meat, fruits and vegetables, fish and spices [22]. Furthermore, according to statistics in 2011, 70 countries and regions worldwide have approved 548 kinds of food and condiments for irradiation treatment [24].

**Figure 2:**
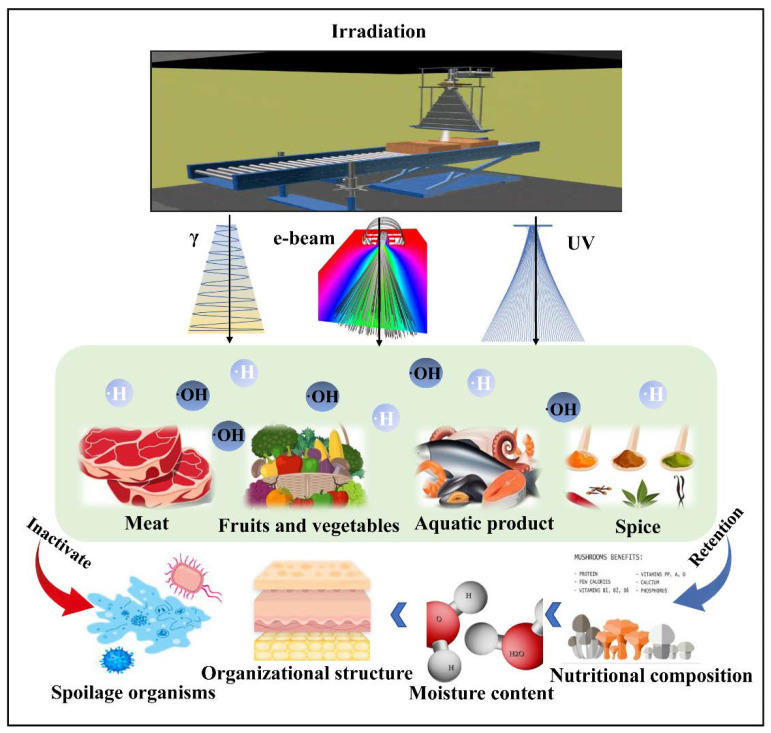
The food system under irradiation technology (γ, gamma radiation; e-beam, electron beam; UV, ultraviolet).

Irradiation preservation mainly uses high-energy radiation to produce active free radicals such as -H and -OH in food (Figure 2). These free radicals interact with nuclear substances to kill pathogenic microorganisms, parasites, etc., inhibit certain metabolic reactions and biological activities in food, delay the deterioration of tissue structure and maintain the sensory and nutritional characteristics, thereby extending the shelf life of foods [25,26]. In particular, common pathogenic bacteria in food such as Salmonella, Escherichia coli O157:H7 and Listeria were inhibited under radiation treatment [27]. In addition, irradiation technology is suitable for the preservation of a variety of edible fungi. This radiation preservation method mainly uses elements with a radioactive effect to process the mushroom body under a certain intensity of rays, and separates the water and other substances in the mushroom body through ionization, which can effectively prevent or reduce the rate of solid own metabolism [28]. Notably, the preservation effect of irradiation on edible fungi depends on the irradiation dose, storage environment, variety and maturity of edible fungi.

### Irradiated Species

Radiation sources, such as gamma irradiation, ultraviolet irradiation and electron beam, are mostly used to cause the effects of ionizing radiation to preserve the quality of edible fungi. At present, irradiation preservation of *Agaricus bisporus* [29], *Volvariella volvacea* [30], *Lentinus edodes* [31], *Pleurotu nebrodensis* [17], *Pleurotus ostreatus* [32], *Laclarius deliciosus* [33], *Flammulina velutipes* [34] and *Pleurotus eryngii* [35] has been studied internationally. Moreover, the recommended dose for extending the shelf life of fresh edible fungi in different countries is 1–3 kGy, while the recommended dose regarding the decontamination of dried edible fungi (included in food additives with spices), used as seasonings, is 10–50 kGy [36]. Table 1 summarizes the parameters of radiation absorbed doses to edible fungi published in the past decade.

Gamma irradiation is electromagnetic waves induced by the release of α or β rays from atomic nuclei. They have strong penetrating and dissociating abilities, do not deflect in the magnetic field, do not generate induced radiation, can cause irradiated molecules to generate paired ions or free radicals and then induce other chemical reactions, especially for products with high moisture content [35]. Currently, there are two internationally approved irradiation sources: ^137^Cs and ^60^Co. Postharvest edible fungi are irradiated by gamma irradiation with strong penetrating power to inhibit the growth of microorganisms, reduce the activity of enzymes and the opening rate and delay the metabolic process of bacteria to achieve the purpose of preservation and storage [21].

Electron beam (e-beam) irradiation involves the use of accelerators that accelerate electrons close to the velocity of light to produce high-energy e-beams. Compared with gamma irradiation, e-beam irradiation has a short processing time, and the irradiation dose (high dose rate) can be controlled by adjusting the transmission speed. In addition, the irradiation process can be initiated and stopped more easily under the control of electric energy. However, unlike gamma rays, the penetration distance of e-beam irradiation is related to the density of the irradiated product. In general, high-energy electrons penetrate over short distances and are more suitable for thin products with regular shapes. It was claimed that an appropriate dose of e-beam irradiation can inhibit the microbial reproduction of edible fungi, delay physiological metabolism and not change their nutritional composition [25,66].

Ultraviolet (UV) irradiation technology is divided into UV-A (400–315 nm), UV-B (315–280 nm) and UV-C (280–100 nm) according to different wavelengths. In recent years, UV-C has been widely used as a substitute for chemical sterilization and microbial reduction in food, and has been approved as a disinfectant for food surface treatment [67]. Pulsed ultraviolet (PUV) light is rich in UV-C light, and could quickly inactivate microbes on surfaces of food with a wavelength range of 180–1100 nm [68]. On the other hand, as a postharvest treatment of fresh produce, a certain dose of UV-C radiation has been shown to reduce fruit and vegetable rot, and delay the senescence and ripening of different fruits and vegetables as well as edible fungi [69].

Overall, the application of gamma, e-beam and UV radiation technology mainly focuses on fresh samples of edible fungi (Table 1 and cited references), but also includes freeze-dried [37] and air-dried samples [64], and is even applied to the aqueous extract of dried edible fungi [38]. The applied doses of gamma and e-beams are around 1 kGy and 2 kGy in most cases. Nevertheless, the dose of UV application varies widely.

## 3. Effects of Irradiation on Edible Fungi

### 3.1. Shelf Life of Edible Fungi

Prolonging shelf life is a key factor for the postharvest commercialization of edible fungi, so as to promote the circulation of edible fungi among regions. Many efforts have been devoted to investigating the shelf life of edible fungi, which is only 1–3 days after harvest at normal temperatures [13]. However, the storage of edible fungi after being irradiated with a dose of a few kGy or less has an obvious effect on extending the shelf life of edible fungi and even various vegetables [36]. In this process, using a radiation dose of 1 kGy at ambient temperature effectively inhibited stalk growth and cap opening of fresh edible fungi, and extended the shelf life [54].

Regarding the effect of irradiation treatment on the shelf life of edible fungi, Wani, Hussain, Meena, Dar and Mir [70] found that a 2.0 kGy dose of gamma radiation can significantly reduce the weight loss of *Agaricus bisporus* and prevent browning and mold growth. Meanwhile, the cap and veil opening of edible fungi was delayed by 9 days, and the shelf life was extended by 12 days. Mami et al. [25] reported that under specified temperature (4 °C) and humidity (80%) storage conditions, the shelf life of *Agaricus bisporus* could be extended to 16 days after irradiation with 2 and 4 kGy e-beam irradiation. Supporting this view, Shi et al. [43] also found that an enhancement in the shelf life of fresh *Lentinula edodes* up to a period of 20 days could be achieved by the application of a gamma ray dose of 1 kGy and storage at 4 °C, and it retained the quality attributes required for its acceptability. Lu et al. [60] studied the shelf life of *Agaricus bisporus* irradiated with short-wavelength ultraviolet rays and it remained relatively stable for 18 days at 4 °C. UV-C irradiation can restrain the ripening of *Agaricus bisporus* and inhibit the activity of polyphenol oxidase during storage, accordingly reducing its respiration rate, diminishing plasmic permeability and delaying browning reaction, thus extending the preservation time. Other studies also revealed that gamma irradiation (1 and 1.5 kGy) in combination with modified atmosphere packaging can extend the storage life of shiitake mushrooms up to 20 days [31]. Similarly, Shi et al. [44] demonstrated that ultrasound combined with gamma ray (1 kGy) treatment could maintain the quality of fresh *Lentinus edodes* during 20 days of storage. These results confirmed that food irradiation may be quite effective, in conjunction with other food preservation techniques such as low temperature or packaging materials, to acquire maximum synergetic effect with minimum process losses. Furthermore, low-dose rate irradiation is beneficial to prolong the shelf life of edible fungi. For instance, under a cumulative irradiation dose (2 kGy), the shelf life of *Agaricus bisporus* could be increased by 2 days when the irradiation dose rate of 4.5 kGy/h was compared with 32 kGy/h [71]. The higher the irradiation dose rate, the less obvious the effect of irradiation on prolonging the shelf life of edible fungi. This may be due to the integrity of the edible fungal cell membrane being seriously damaged by the high dose rate, which changes the permeability of the cell membrane, resulting in the attenuation of the edible fungal cell defense function and the invasion of external harmful substances [27,72].

### 3.2. Pathogenic Microorganisms of Edible Fungi

Microbial infection is the main reason for the spoilage of edible fungi. Combined with their rich nutrients and moisture content, edible fungi provide favorable conditions for the growth and reproduction of microorganisms. Different studies have indicated that some pathogenic bacteria are particularly sensitive to irradiation treatment [27]. In general, the principle of inactivation of microorganisms by irradiation has two parts: on one hand, ionizing radiation (UV) inhibits the replication and transcription of genetic material [73]. On the other hand, ionizing radiation (such as gamma ray or e-beam) directly acts on biological molecules, breaking hydrogen bonds, oxidizing double bonds, destroying ring structures or polymerizing some molecules, changing the structure of biological macromolecules [70,74]. In addition, microorganisms irradiated by high-energy electron rays will ionize, hindering all activities in the microbial cells and leading to microbial cell death. Meanwhile, different radiation doses and electron energies are needed to control different microorganisms. The main studies regarding the impact of irradiation on edible fungal microorganisms published in the past decade are summarized in Table 2.

In terms of irradiation sterilization, recent studies have found that irradiation treatment can cause qualitative and quantitative changes in the microbial flora of edible fungi. It can reduce the total plate count, coliform group count, psychrophilic bacterial count and yeast and mold count to a certain extent, and obtain valuable results to ensure the quality of fresh edible fungi. Dong et al. [29] found that an e-beam irradiation dose of 1 kGy and above effectively inhibits the growth and reproduction of *Pseudomonas* during storage of *Agaricus bisporus*. These results were in agreement with Yurttas et al. [54] who showed that e-beam irradiation treatment (1 kGy) had a significant impact on reducing *aerobic* and *psychrotrophic* populations, whereas higher doses were required for inhibition of *yeasts and molds*.

Generally, the combination of different preservation technologies can maintain the product quality required during processing and storage as much as possible. Guan, Fan and Yan [55] treated *Agaricus bisporus* with hydrogen peroxide and found that UV-C could reduce the value of *E. coli* O157:H7 and the total number of colonies, compared with the control group. A recent study by Jiang, Luo, Chen, Shen and Ying [31] indicated that 2 kGy gamma irradiation combined with modified atmosphere packaging can effectively reduce the microbial content (*Pseudomonas*, *psychrophilic*, *mesophilic*, *yeasts and molds*) in *Lentinus edodes*. Additionally, the combination of ultrasound and gamma irradiation treatment had the best synergistic effects on the inhibition of microbial growth during storage [44]. Ramos Villarroel [75] found that the initial counts of Escherichia coli and Listeria monocytogenes inoculated on fresh-cut mushrooms (*Agaricus bisporus*) were reduced by 3 and 2 logarithms, respectively, after UV pulse treatment of a full wavelength spectrum (180–1100 nm), indicating that there was antibacterial effect in this spectral range. These results demonstrated that the conversion of radiation technology has the ability to inactivate microorganisms without compromising food quality, as well as being a greener and safer technology. Therefore, in order to avoid the deterioration of edible fungi during storage and to ensure safety regarding their consumption, irradiation of edible fungi may be the best possible option.

### 3.3. Sensory Quality of Edible Fungi

Regarding the effects of irradiation technology on the sensory quality of edible fungi, changes in flavor, texture and color have been reported by some researchers. It was previously reported by Mau and Hwang [76] that the volatile flavor components of fresh edible fungi were mainly composed of eight-carbon compounds, including 1-octanol, 3-octanol, 3-octanone, 1-octen-3-ol, 2-octen-1-ol and 1-octen-3-one. Among them, 1-octene-3-ol was the most important compound related to the flavor of fresh edible fungi, and the content would decrease with the extension of the postharvest storage period. In addition, Akram et al. [77] found that irradiation with 1 kGy increased the eight-carbon volatile components of fresh shiitake, while 2 and 3 kGy irradiation treatment of fresh shiitake produced some new volatile compounds, such as methylethyl disulfide and sulfinylbis methane. However, the eight-carbon compounds mostly disappeared after drying. It is notable that gamma ray irradiation (5 kGy) of dried *Agaricus bisporus* reduced the total volatile compounds by more than 50%, and there was no flavor difference between the irradiated and non-irradiated samples in sensory evaluation [12].

Texture is the main quality measure for consumers when evaluating the freshness of edible fungi [45]. Overall, the firmness of fruits and vegetables mainly depends on the mechanical strength of the cell wall and the expansion pressure of the cell [10]. Table 3 summarizes the changes in texture and color of edible fungi by irradiation technology in recent years. Notably, the effect of irradiation treatment on the sensory quality of edible fungi was closely related to the irradiation dose. Several researchers have found better retention of texture/higher firmness or delay in postharvest edible fungi softening with low-dose e-beam or gamma irradiation. For instance, Dong et al. [29] found that the firmness of mushrooms in the treated group dramatically exceeded other groups under low-dose (1 kGy) e-beam irradiation. Duan, Xing, Shao and Zhao [66] showed that softening was delayed by irradiating freshly harvested mushrooms with different doses of e-beams. Among them, the dose of 2 kGy could inhibit the respiration of mushrooms, reduce the weight loss rate and maintain the turgor pressure of cells and a better firmness. Furthermore, Jiang, Luo, Chen, Shen and Ying [31] indicated that a 1 kGy gamma irradiation dose effectively maintained the firmness of edible fungi, while there was a negative impact on texture, chemical properties and functional components at a higher dose (2 kGy). Hou et al. [46] also reported that 0.8 kGy gamma irradiation slowed down the weight loss and softening of *Volvariella volvacea*. The softening of edible fungi during storage may be related to the degradation of the cell wall and microbial infection. Moreover, *Pseudomonas* can destroy the intracellular matrix and make the central vacuole smaller or even disappear, reducing the mechanical strength of the cell wall and the turgor pressure of the cell, resulting in partial collapse of the cell and loss of turgor pressure [78]. This softening phenomenon of edible fungi caused by microbial infection has been observed in untreated groups, but irradiation treatment can effectively delay the softening process and still maintain good firmness in the later storage period. In addition, Xiong, Xing, Feng, Tan and Bian [17] combined refrigeration with low-dose (1.2 kGy) gamma radiation and applied it to edible fungi. The treatment group had significantly delayed occurrence of softening, splitting and browning of fruiting bodies (6–9 days), while the loss of texture was minimized compared with refrigeration treatment alone.

The color of edible fungi is an intuitive impression for consumers to judge the quality attribute. Browning is the main reason for the quality loss of edible fungi [14]. This is mainly caused by two reasons: for one thing, it is an extremely complex chemical reaction between various oxygenated compounds such as organic acids, carbohydrates and amino acids, also known as non-enzymatic browning. The toxic substances and odor are produced slowly from the browning of edible fungi [18]. Secondly, enzymatic browning can occur. The browning substrate in plant cells is an acid substance that can react in the presence of oxygen and phenolic oxidase to oxidize brown or red quinones. This is also the main reaction causing browning of edible fungi [71,79]. Regarding the effect of irradiation technology on the color of edible fungi, all the authors reported the conclusion that browning was delayed. For example, longer retention of whiteness and/or color improvement was also confirmed in irradiated *Agaricus bisporus* [25,45], *Lactarius deliciosus* [33], *Lentinula edodes* [44,64] and *Volvariella volvacea* [46] samples. Shiitake mushrooms pretreated with pulses of UV light reduced the initial browning index and browning degree values by 43.02% and 47.54%, respectively, improving the surface color of dried shiitake mushrooms [80]. This was probably due to irradiation diminishing enzymatic browning by inhibiting polyphenol oxidase activity in edible fungi [81]. Other previous studies also showed that *Agaricus bisporus* exposed to UV-C irradiation had more severe browning with the increase in dose (0.45 kJ/m^2^) [55].

### 3.4. Nutritional Composition of Edible Fungi

Edible fungi are nutritionally rich and a good source of carbon and nitrogen. Changes in composition have a great impact on product quality and nutritional health value. The effects of irradiation on nutrients of edible fungi, including proteins, fat, sugars and vitamins, were described by different authors in several edible fungi species in the past decade and are summarized in Table 3.

Concerning the effect of irradiation technology on the soluble protein of edible fungi, some studies have confirmed that *Agaricus bisporus* (4 kGy) [25], *Amanita* (10 kGy) [50] and *Boletus pinophilus* (2 kGy) [41] treated with irradiation exhibited smaller initial declines in soluble protein content than non-irradiated controls, while higher doses showed negative effects [40]. Possibly, the higher sensitivity of proteins was related to the scission of the C\N bonds in the backbone of the polypeptide chain or splitting of the disulfide bonds, or physical changes such as unfolding or aggregation. Dawoud and Abu-Taleb [82] found that protein and free amino acid contents did not drop after using 0.5 kGy gamma irradiation to irradiate *Pleurotus ostreatus*. As reported by Jiang, Luo, Chen, Shen and Ying [31], the soluble protein content of shiitake mushroom samples treated with gamma rays during storage (the initial level was 54.1%) decreased slightly, while the decrease rate during this period increased at higher doses (2 kGy). Nevertheless, Wang, Chu and Kou [57] showed that the soluble protein content of *Pleurotus ostreatus* remained basically unchanged after 4.0 kJ/m^2^ UV-C irradiation.

For fatty acids, some researchers investigated the effect of irradiation technology on the nutrients of edible fungi. Cardoso et al. [49] confirmed that irradiation maintained the content of polyunsaturated fatty acids (PUFAs) in dried edible fungi between 81% and 82%, saturated fatty acids (SFAs) only accounted for 12% to 34% and monounsaturated fatty acids (MUFAs) were less than 2%. Remarkably, the high incidence of PUFA indicates that edible fungus powder contains healthy unsaturated fats, which also helps edible fungus powder to be regarded as a healthy food. Palmitic acid, oleic acid and linoleic acid are the main fatty acid composition of edible fungi, which is related to the acceptable organoleptic properties of edible fungi. Among them, linoleic acid is the precursor of the aroma component of edible fungi [11]. Some researchers have reported that both gamma and e-beam irradiation can change the fatty acid profiles of edible fungi [39,48,50,51,53]. In this way, saturated fatty acids seem to be more resistant to radiation, while PUFA is more sensitive to radiolysis than MUFA, thereby the percentage tends to decrease [40]. The changes in lipid profiles may be divided into two situations, one is the auto-oxidation of fatty acids and molecular oxygen by radiation as catalysis, and the other is the self-effect of high-energy radiation [83]. Obviously, the unsaturated molecules of fatty acids were changed after irradiation. The general mechanism of lipid radiolysis is mainly primary ionization, followed by the migration of a positive charge to the carboxyl carbonyl group or double bond, leading to the degradation of unsaturated fatty acids [73]. However, this phenomenon mostly occurs in fresh edible fungi. In addition, Fernandes et al. [39] observed that the change in fat content of *Boletus edulis* during storage was affected in non-irradiated and high-dose irradiated samples (10 kGy), while relatively lower doses (2 or 6 kGy) kept fat levels more stable during storage.

Free sugars are one of the most important parameters in quality assessment and are considered a good indicator of suitable conservation techniques due to their sensitivity to technical practices [84]. Concomitantly, radiation causes changes such as melting point decreases, reduction in optical rotation and browning of sugars. The free sugars that can be detected in edible fungi are mainly trehalose, mannitol and fructose. Cardoso et al. [49] found that the total sugar content of the irradiated *Agaricus bisporus* decreased gradually within 12 months, while the trehalose content increased, but there was no significant correlation with the storage period. This is consistent with that reported by Fernandes et al. [51] and Fernandes et al. [40] who observed that the sugar content of irradiated edible fungi changed significantly. In this process, the sugar may be degraded, producing a particular atmosphere mainly composed of H_2_ and CO_2_, as well as traces of CH_4_, CO and H_2_O [73]. Likewise, Fernandes et al. [50] reported that the sugar content of edible fungi had a significant change after e-beam irradiation, but the effects of the two edible fungus species were not consistent, namely, the sugar content increased with irradiation in *A. caesarea*, but not in *A. curtipes*. However, the sugar content of irradiated *Macrolepiota procera* did not change significantly during storage [53].

Edible fungi are also a good resource of vitamins, being rich in vitamins B, C, D and E. Regarding the effects of irradiation on the vitamins of edible fungi, some researchers have confirmed that the total tocopherol content of edible fungi irradiated by gamma rays [40,41] or e-beam [50,51,53] tended to be higher than that of the non-irradiated controls, while showing the opposite result at high doses. Furthermore, unlike gamma and e-beam irradiation, the main role of UV irradiation in the edible fungus industry is to increase their vitamin D content. For example, irradiating fresh *Agaricus bisporus* and freeze-dried *Hygrophorus marzuolus* with UV-C increased their vitamin D up to 14-fold and 9-fold, respectively [85]. Similar results were found in the study of Guan et al. [56]. In addition, Huang et al. [65] observed that the content of vitamin D_2_ in the fruiting bodies of 11 fresh edible fungi significantly increased from 0–3.93 to 15.06–208.65 mg/g when irradiated with UV-B. Meanwhile, Simon, Phillips, Horst and Munro [86] showed that UV-B irradiated *Agaricus bisporus* showed no significant effect regarding the change in water-soluble vitamin content. At present, the European Commission has formulated regulations on the exposure of *Agaricus bisporus* to ultraviolet light (UV) irradiation to induce the conversion of provitamin D-2 (ergosterol) into vitamin D-2 (ergocaciferol) content, ensuring the safety of mushroom powder as a novel food [87].

### 3.5. Enzymatic Activity of Edible Fungi

**Figure 3:**
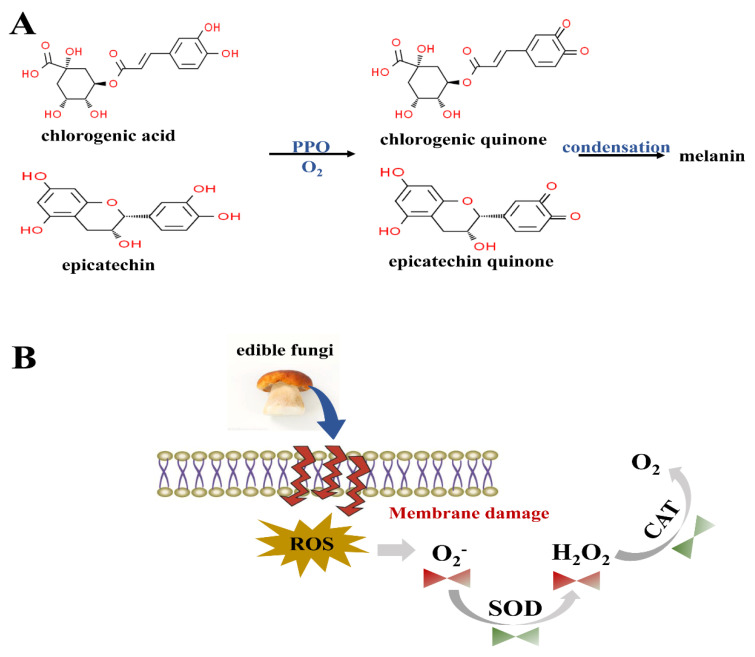
The mechanism of polyphenol oxidase (**A**) and antioxidant enzyme (**B**) in edible fungi. (PPO, polyphenol oxidase; SOD, superoxide dismutase; CAT, catalase).

Irradiation has a great impact on the activities of various enzymes in edible fungi, such as polyphenol oxidase (PPO), phenylalanine ammonia-lyase (PAL), catalase (CAT) and superoxide dismutase (SOD), which is summarized in Table 3. Dong et al. [29] applied e-beam irradiation to decelerate the increase in PPO content of *Agaricus bosporus* during storage, as compared to the non-irradiation group. In fact, the mechanism of enzymatic browning of edible fungi is that PPO catalyzes the oxidation of monophenols (epicatechin and chlorogenic acid) to o-quinones (epicatechin quinone and chlorogenic quinone), which leads to the formation of brown spots (melanin) (Figure 3A). In this process, e-beam irradiation treatment inactivated the PPO activity of *Agaricus bisporus* during postharvest storage and inhibited the browning phenomenon. Likewise, the activities of SOD and CAT of *Agaricus bisporus* treated with e-beams were always higher than those of the control group throughout the storage period, with the most pronounced effect after treatment with 1.0 kGy. SOD and CAT are the key components of the cellular antioxidant defense system, which can protect cells from the attack of reactive oxygen species and maintain the freshness of food by protecting the integrity of the cell membrane (Figure 3B) [17]. Nie et al. [61] showed that the combined treatment of UV-C irradiation and biocontrol yeast (*Pichia kluyveri*) induced the activity levels of SOD and CAT in mushrooms to be higher than in untreated (control) mushrooms. A similar result was also found in studying the effect of gamma irradiation on the enzyme activity of *Volvariella volvacea* by Hou et al. [46]. In addition, Shi et al. [44] showed that the PAL activity level of *Lentinula edodes* combined with gamma and ultrasound was higher than the control group throughout refrigeration storage, except on days 4 and 20. In general, PAL is directly related to the synthesis of phenolic compounds in cells, and is also one of the antioxidant enzymes involved in the synthesis of secondary metabolites [88]. On the other hand, the increase in PAL activity contributes to the accumulation of phenols, forming phenolic compounds such as flavonoids and enhancing phenolic retention during late storage. Meanwhile, Wang et al. [59] also reported that the PAL content of oyster mushrooms treated by UV-C irradiation was relatively high during the storage period, except on day 0. These results demonstrate that an appropriate radiation dose reduces the activity of related enzymes and promotes the accumulation of phenolic compounds.

## 4. Conclusions and Future Perspectives

In the current review, the research progress of postharvest radiation preservation technology for edible fungi focuses on the types of radiation preservation technology and radiation effects, including the effects of radiation on the shelf life, microorganisms, sensory quality, nutritional components and enzyme activities of edible fungi. In general, with the development of the edible fungus industry and the increase in the output of edible fungi, the radiation preservation technology of edible fungi belongs includes physical sterilization technology and is a promising method for the preservation of edible fungi, having a good sterilization effect, no pollution, no residue, low energy consumption and maintaining the sense and quality of edible fungi to the greatest extent. Indicatively, from a global point of view, 1–3 kGy irradiation doses are recommended for fresh edible fungi, and doses below 10 kGy are recommended for dried edible fungi. It is noteworthy that the irradiation technology may affect the nutritional composition of edible fungi to varying degrees, according to the species of edible fungus, the irradiation dose and the types of irradiation source. Furthermore, postharvest internal quality degradation of edible fungi is inevitable, and the current research on the effect of radiation-induced flavor differences is still in its infancy, therefore further in-depth research is needed. On the other hand, some novel technologies should be encouraged, such as the combined use of irradiation technology and other preservation treatment technologies, and combining different technologies with lower capital cost or shorter processing time to further improve the postharvest preservation effect of edible fungi.

## Figures and Tables

**Table 1:** Irradiated edible fungal species and irradiation conditions.

Radiation Source	Species	Origin	Sample	Doses	Reference
γ radiation	*Macrolepiota procera*	Portugal	Freeze-dried/Fresh	0.5 and 1 kGy	[37]
	*Agaricus bisporus* (extract β-glucan)	India	Dried (oven)	5, 10, 20, 30 and 50 kGy	[38]
	*Boletus edulis*	Portugal	Dried and sliced	2, 6 and 10 kGy	[39]
	*Lactarius deliciosus*	Portugal	Fresh	0.5 and 1 kGy	[33]
	*Boletus edulis, Hydnum repandum*	Portugal	Fresh	1 and 2 kGy	[40]
	*Boletus pinophilus, Clitocybe subconnexa*	Portugal	Fresh	2 kGy	[41]
	*Lentinus edodes*	Korea	Dried	2, 5 and 10 kGy	[42]
	*Lentinula edodes*	China	Fresh	1 and 2 kGy	[43]
	*Lentinula edodes*	China	Fresh	1 kGy	[44]
	*Agaricus bisporus*	Iran	Fresh	1 and 2 kGy	[45]
	*Volvariella volvacea*	China	Fresh	0.2, 0.4, 0.6, 0.8 and 1.0 kGy	[46]
γ and e-beam radiation	*Macrolepiota procera*	Portugal	Dried (oven)	0.5, 1 and 6 kGy	[47]
	*Agaricus bisporus*	Portugal	Fresh	1, 2 and 5 kGy	[48]
	*Agaricus bisporus*	Portugal	Dried (oven)	1, 2, 5 and 10 kGy	[49]
E-beam	*Agaricus bisporus*	China	Fresh	0.5, 1, 1.5 and 2 kGy	[29]
	*Agaricus bisporus*	Iran	Dried	0.5, 1, 2 and 4 kGy	[25]
	*Amanita* (*A. caesarea* and *A. curtipes*)	Portugal	Dried (oven)	2, 6 and 10 kGy	[50]
	*Boletus edulis, Russula delica*	Portugal	Dried (oven)	2, 6 and 10 kGy	[51]
	*Boletus edulis, Macrolepiota procera*	Portugal	Dried (oven)	2, 6 and 10 kGy; 0.5, 1 and 6 kGy	[52]
	*Macrolepiota procera*	Portugal	Dried (oven)	0.5, 1 and 6 kGy	[53]
	*Agaricus bisporus*	USA	Dried and sliced	1 kGy	[54]
UV-C	*Agaricus bisporus*	China	Fresh	0.45 kJ/m^2^	[55]
	*Agaricus bisporus*	China	Freeze-dried	1 kJ/m^2^	[14]
	*Agaricus bisporus*	China	Freeze-dried	0.5, 1 and 2 kJ/m^2^	[56]
	*Pleurotus ostreatus*	China	Fresh	4 kJ/m^2^	[57]
	*Agaricus bisporus*	Canada	Dried	37.8, 56.7 mJ/cm^2^	[58]
	*Agaricus bisporus*	China	Fresh	0.5 to 9 kJ/m^2^	[59]
	*Agaricus bisporus*	China	Fresh	0.5, 1 and 2 kJ/m^2^	[60]
	*Pleurotus eryngii*	China	Fresh	2, 4 and 6 kJ/m^2^	[61]
UV-C and UV-A	*Pleurotus ostreatus*	Kenya	Fresh	1.96 and 0.21 kJ/m^2^	[62]
UV-B	*Lentinula edodes*	Korea	Fresh	30 kJ/m^2^	[63]
	*Lentinula edodes*	India	Air-dried	0.5, 1, 1.5, 2 and 2.5 h	[64]
	*Agaricus blazei, Agrocybe cylindracea, Auricularia polytricha, Hypsizigus marmoreus, Lentinula edodes, Pholiota nameko, Pleurotus eryngii, Pleurotus citrinopileatus, Pleurotus ferulae, Pleurotus ostreatus, Pleurotus salmoneostramineus*	China	Fresh	2 h, 25.9 kJ/m^2^	[65]

E-beam: Electron beam; UV: Ultraviolet.

**Table 2:** Effects of radiation on the inhibition of pathogenic microorganisms of edible fungi.

Species	Radiation Source	Time for Analysis	Parameter	Results	Reference
*Agaricus bisporus*	E-beam	Every 3 days until 21 days	*Pseudomonas* content	Irradiation levels above 1 kGy inhibited the growth and reproduction of *Pseudomonas*.	[29]
*Agaricus bisporus*	γ radiation	Every 7 days until 21 days	*Escherichia coli* (*E. coli*), *Staphylococcus aureus* (*S. aureus*)	Dose of 2 kGy and Ag nanoparticle polyethylene films could prevent the accumulation of microbial load.	[45]
*Lentinula edodes*	γ radiation	Every 4 days until 20 days	colonies, *Pseudomonas*, *molds and yeasts*, *Enterobacteriaceae*	Ultrasound and gamma irradiation (1 kGy) reduced natural microflora present, such as the total number of colonies, molds, yeasts, *Pseudomonas* and *Enterobacteriaceae*.	[44]
*Agaricus bisporus*	E-beam	0, 4, 9, 12, 15 days	Total *aerobic* plates, *psychrotrophic*, and *yeast and mold* counts	E-beam irradiation had an impact on reducing *aerobic* and *psychrotrophic* populations, whereas higher doses were required for inhibition of *yeasts and molds*.	[54]
*Pleurotus ostreatus*	UV radiation	Every 3 days until 15 days	Total viable count, *lactobacillus*, *yeasts and mold*	UV-C radiation (4 kJ/m^2^) delayed the growth of microbiological population of oyster mushrooms during the storage period.	[57]
*Pleurotus eryngii*	UV radiation	Every day until 4 days	*Lactococcus lactis* subsp.	The combination of UV-C and yeast (*Pichia kluyveri*) significantly increased the level of decay control on mushrooms than either treatment alone.	[61]
*Agaricus bisporus*	UV radiation	15 days	*Listeria* bacteriophages	The growth of *L. monocytogenes* was inhibited on mushrooms treated with chitosan, electrolyzed water, peroxyacetic acid or UV.	[58]
*Agaricus bisporus*	UV radiation	Every 7 days until 14 days	*Escherichia coli* O157:H7, microbial loads	The inactivation effect of H_2_O_2_+UV on *E. coli* O157:H7 inoculated on mushrooms was not obvious, but reduced the microbial loads and extended the shelf life.	[55]
*Volvariella volvacea*	γ radiation	Every day until 7 days	bacteria count, *yeasts and mold*	The results showed that V. volvacea exposed to 60Co gamma irradiation resulted in decreased total bacterial, mold and yeast counts throughout storage compared with that of the non-irradiated control.	[46]

E-beam, Electron beam; UV radiation, Ultraviolet radiation.

**Table 3:** Effects of irradiation on physico-chemical, biochemical parameters and nutritional properties of edible fungi.

Species	Radiation Source	Time for Analysis	Texture	Color	Nutrients	Enzymatic Activity	Reference
*Agaricus bisporus*	E-beam	Every 3 days until 21 days	The firmness of irradiated group dramatically exceeded non-irradiated samples, and the texture was improved.	The increase inbrowning index was inhibited.	/	Irradiation group decreased PPO activity, while increased SOD and CAT activity.	[29]
	γ radiation	Every 7 days until 21 days	The firmness and elasticity of the combined treatment (2 kGy) were higher than non-irradiated samples.	Combined treatment (2 kGy) kept the color stable.	/	/	[45]
	E-beam	Every 4 days until 16 days	/	Irradiation with 1 and 2 kGy appeared to produce higher L* value than non-irradiated samples.	The protein content of irradiated group was the highest and increased with the increase in dose (4 kGy). However, this treatment reduced the content of vitamin C.	/	[25]
	γ radiation, e-beam	0, 6, 12 months	/	/	E-beam or gamma rays (up to a dose of 10 kGy) all benefited the preservation of specific nutrients such as sugars, fat, tocopherols and organic acids.	/	[49]
	E-beam, UV radiation	Every 4 days until 8 days	/	/	In general, irradiation did not cause marked changes in protein and tocopherol contents, except for a small number of organic acids and saturated fatty acids.	/	[48]
	UV radiation	Every 6 days until 18 days	/	UV-C irradiation suppressed browning during cold storage.	/	/	[60]
*Lentinula edodes*	γ radiation, e-beam	Every 4 days until 20 days	/	Ultrasound and gamma irradiation (1 kGy) maintained a better appearance.	/	Treatment group increased PAL activity.	[44]
	UV radiation	0.5, 1.0, 1.5, 2.0 and 2.5 h	/	With the increase in the irradiation duration, the L* value of the samples decreased, the b* value had no significant difference, while the a* values depicted a slight increase.	The content of vitamin D_2_ was increased in irradiated samples (2 h), while the amino acid content did not change significantly.	/	[64]
	UV radiation	/	The hardness of UV-reated was slightly increased compared to that of untreated samples. However, there were no significant differences in all textural indices.	There were no statistically significant changes in L*, a* or b* value between UV-treated and untreated groups.	-	/	[63]
*Macrolepiota procera*	γ radiation	/	/	/	Irradiation treatment did not cause marked changes in mushrooms’ nutritional composition, but combining freezing treatment with 0.5 kGy doses preserved total tocopherols.	/	[37]
	E-beam	0, 6 and 12 months	/	/	In general, irradiation treatment did not cause significant changes in nutrients (carbohydrate, total sugar and protein), while this treatment changed the distribution of the fatty acids and reduced the tocopherol content.	/	[53]
*Volvariella volvacea*	γ radiation	Every day until 7 days	All samples softened as the storage time increases. The firmness of the samples irradiated with 0.8 kGy of gamma radiation changed the least over the storage period.	The degree of browning increased initially, then decreased and finally increased again during the storage period, while the percentage of browning in the control group was significantly higher than those of the gamma-irradiated samples.	/	Irradiation group (0.8 kGy) increased the CAT activity and SOD activity compared with the control group.	[46]
*Lactarius deliciosus*	γ radiation	Every day until 8 days	/	Compared with the non-irradiated group, there was a slight decrease in a* value with irradiation dose.	/	/	[33]
*Boletus edulis*	γ radiation	0, 6 and 12 months	/	/	The obtained profiles presented lower amounts of fat and proteins, and higher amounts of carbohydrates. However, the nutrition content of the irradiated group was stable for long periods.	/	[39]
*Boletus edulis, Hydnum repandum*	γ radiation	/	/	/	Irradiation treatment decreased the protein, total sugar content and unsaturated fatty acids. Meanwhile, it increased the total tocopherol content.	/	[40]
*Boletus edulis, Russula delica*	E-beam	/	/	/	The contents of protein and total sugar were decreased in the irradiation group. However, irradiation changed fatty acid distribution and increased tocopherol content.	/	[51]
*Amanita* (*A. caesarea* and *A. curtipes*)	E-beam	/	/	/	Overall, irradiation treatment increased the contents of protein and tocopherol, but changed the distribution of fatty acids. In addition, the total sugar content increased in irradiated *A. caesarea* and decreased in irradiated *A. curtipes*.	/	[50]
*Pleurotus ostreatus*	UV radiation	Every 3 days until 15 days	/	Irradiation prevented the browning of mushroom surface, and the L* value of untreated samples was significantly lower compared with UV-C treatment.	The change in soluble protein content was similar between untreated and irradiated samples, but the content of irradiated samples was significantly higher than that of untreated samples during entire storage period.	UV-C irradiation increased the PAL activity.	[57]
*Boletus pinophilus, Clitocybe subconnexa*	γ radiation	/	/	/	The radiation treatment group increased the protein content and decreased the total sugar and tocopherol content.	/	[41]
*Pleurotus eryngii*	UV radiation	Every day until 4 days	/	/	/	UV-C irradiation increased the CAT and SOD activity.	[61]

E-beam, electron beam; UV radiation, ultraviolet radiation.; L*: lightness; a*: redness; b*: yellowness; PPO: polyphenol oxidase; PAL: phenylalanine ammonia-lyase; CAT: catalase; SOD: superoxide dismutase./: Not reported.
